# Single-Cell Transcriptomic Approaches for Decoding Non-Coding RNA Mechanisms in Colorectal Cancer

**DOI:** 10.3390/ncrna11020024

**Published:** 2025-03-10

**Authors:** Mahnoor Naseer Gondal, Hafiz Muhammad Umer Farooqi

**Affiliations:** 1 Department of Computational Medicine & Bioinformatics, University of Michigan, Ann Arbor, MI 48109, USA; gondal@umich.edu; 2 Michigan Center for Translational Pathology, University of Michigan, Ann Arbor, MI 48109, USA; 3 Laboratory of Energy Metabolism, Division of Metabolic Disorders, Children’s Hospital of Orange County, Orange, CA 92868, USA

**Keywords:** single-cell sequencing, colorectal cancer, non-coding RNA, bioinformatics, therapeutics

## Abstract

Non-coding RNAs (ncRNAs) play crucial roles in colorectal cancer (CRC) development and progression. Recent developments in single-cell transcriptome profiling methods have revealed surprising levels of expression variability among seemingly homogeneous cells, suggesting the existence of many more cell types than previously estimated. This review synthesizes recent advances in ncRNA research in CRC, emphasizing single-cell bioinformatics approaches for their analysis. We explore computational methods and tools used for ncRNA identification, characterization, and functional prediction in CRC, with a focus on single-cell RNA sequencing (scRNA-seq) data. The review highlights key bioinformatics strategies, including sequence-based and structure-based approaches, machine learning applications, and multi-omics data integration. We discuss how these computational techniques can be applied to analyze differential expression, perform functional enrichment, and construct regulatory networks involving ncRNAs in CRC. Additionally, we examine the role of bioinformatics in leveraging ncRNAs as diagnostic and prognostic biomarkers for CRC. We also discuss recent scRNA-seq studies revealing ncRNA heterogeneity in CRC. This review aims to provide a comprehensive overview of the current state of single-cell bioinformatics in ncRNA CRC research and outline future directions in this rapidly evolving field, emphasizing the integration of computational approaches with experimental validation to advance our understanding of ncRNA biology in CRC.

## 1. Introduction

**Figure 1:**
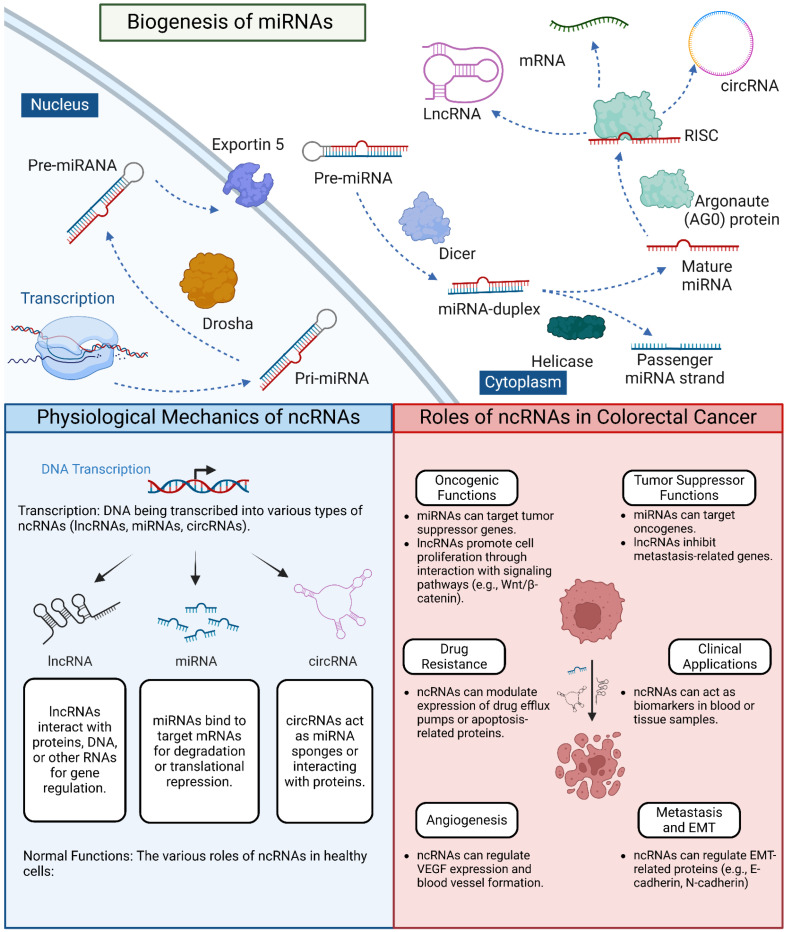
Biogenesis and Functions of Non-Coding RNAs in Cellular Processes and Colorectal Cancer. This figure illustrates the biogenesis of microRNAs (miRNAs) and the roles of various non-coding RNAs (ncRNAs) in normal cellular processes and colorectal cancer (CRC) progression. The top panel depicts the miRNA biogenesis pathway, from transcription to RISC complex formation, and the interactions between different types of ncRNAs. The left panel depicts normal ncRNA functions, including transcription, and regulatory activities. The right panel shows how these processes are dysregulated in CRC, highlighting oncogenic functions, tumor suppressor roles, and involvement in metastasis, angiogenesis, and drug resistance.

Colorectal cancer (CRC) is a complex and heterogeneous disease characterized by the accumulation of genetic and epigenetic alterations that lead to uncontrolled cell growth and metastasis [1,2,3]. While research has traditionally focused on protein-coding genes, it is now clear that the non-coding portion of the genome plays a crucial role in CRC biology. Non-coding RNAs (ncRNAs), including long non-coding RNAs (lncRNAs) and microRNAs (miRNAs), have emerged as critical regulators of gene expression and cellular processes in CRC, influencing various aspects of tumor development and progression [4,5,6] (Figure 1). The rapid advancement of high-throughput sequencing technologies, particularly single-cell RNA sequencing, has led to an explosion of data on ncRNAs in CRC, necessitating sophisticated bioinformatics approaches for their analysis and interpretation [7,8,9]. These computational methods have become indispensable in uncovering the complex roles of ncRNAs in CRC initiation, progression, and metastasis at unprecedented resolution. Single-cell analysis has revealed significant heterogeneity in ncRNA expression patterns within CRC tumors, highlighting the importance of studying these molecules at the cellular level [10].

Recent scRNA-seq studies have provided groundbreaking insights into ncRNA heterogeneity in CRC. For instance, Mihaljevic et al. analyzed 49,436 single cells from 29 CRC patients, demonstrating that lncRNAs exhibit significantly higher cell type specificity compared to protein-coding genes. This study identified 996 lncRNAs strongly enriched in epithelial cells, with 98 of these differentially expressed in tumor samples compared to normal controls [10]. Such findings underscore the potential of targeting cell type-specific ncRNAs as a novel therapeutic strategy in CRC. In this review, we provide a comprehensive overview of the current single-cell bioinformatics strategies employed in ncRNA CRC research. We explore the computational methods and tools used for ncRNA identification, characterization, and functional prediction in CRC at the single-cell level. The review highlights key bioinformatics approaches, including sequence-based methods (e.g., BLAST, INFERNAL), structure-based methods (e.g., RNAfold, IPknot), and machine learning applications (e.g., DeepBind, lncRNAnet) [11,12,13]. We examine how these computational techniques can be applied to analyze differential expression, perform functional enrichment, and construct regulatory networks involving ncRNAs in CRC, with a focus on cell type-specific patterns. Tools like MOFA+ [14] enable the integration of scRNA-seq data with other omics data types, while methods such as MAST and scDEA account for the unique characteristics of single-cell data in differential expression analysis [15,16]. Additionally, we explore the role of single-cell bioinformatics in leveraging ncRNAs as diagnostic and prognostic biomarkers for CRC, as well as in the development of ncRNA-based therapeutic strategies.

This review aims to synthesize the current state of single-cell bioinformatics in ncRNA CRC research and outline future directions in this rapidly evolving field. We emphasize the integration of computational approaches with experimental validation to advance our understanding of ncRNA biology in CRC, with the ultimate goal of improving diagnosis, prognosis, and treatment of CRC. By highlighting the latest advancements in single-cell analysis of ncRNAs in CRC, we provide a comprehensive resource for researchers and clinicians working towards personalized medicine approaches in colorectal cancer management.

## 2. Non-Coding RNAs in Colorectal Cancer

### 2.1. Key ncRNAs Involved in CRC Progression

Several non-coding RNAs have been identified as key players in colorectal cancer progression. Long non-coding RNAs such as HOTAIR, MALAT1, and H19 have been implicated in CRC metastasis and poor prognosis [4,17,18,19]. HOTAIR promotes CRC progression by regulating chromatin state and gene expression. MicroRNAs (miRNAs) also play crucial roles, with miR-21 and miR-31 frequently upregulated in CRC, promoting cell proliferation and invasion. Conversely, miR-145 and miR-143 are often downregulated and act as tumor suppressors [20,21,22]. Circular RNAs (circRNAs) are emerging as important regulators in CRC, with circCCDC66 and circHIPK3 shown to promote CRC growth and metastasis [23,24,25]. The details of ncRNA classification can be found in Table 1.

### 2.2. Roles of ncRNAs in CRC Hallmarks

Non-coding RNAs contribute to various hallmarks of cancer in CRC. They regulate sustained proliferative signaling, with lncRNA CCAT2 promoting MYC expression and cell proliferation [43,44]. In evading growth suppressors, miR-135b targets APC, a key tumor suppressor in CRC [21,45]. NcRNAs also influence resistance to cell death, exemplified by lncRNA TUG1 inhibiting apoptosis through p53 regulation [46,47]. In enabling replicative immortality, TERRA ncRNAs regulate telomere maintenance [48]. Angiogenesis is modulated by ncRNAs like miR-126, which targets VEGF signaling [49,50]. Activation of invasion and metastasis involves ncRNAs such as lncRNA FEZF1-AS1, which promotes epithelial–mesenchymal transition [51,52,53].

Recent studies have uncovered additional roles of ncRNAs in CRC progression. miR-34a has been implicated in chemoresistance, with low expression associated with oxaliplatin resistance and overexpression enhancing chemosensitivity by targeting the SIRT1 and TGF-β/Smad4 pathway [54]. The lncRNA NALT1 promotes CRC proliferation, migration, and invasion by sponging miR-574-5p to upregulate PEG10, with high expression correlated with advanced cancer stage [55]. Circular RNA circ_001569 has been shown to promote CRC proliferation and invasion by sponging miR-145 to regulate E2F5, BAG4, and FMNL2 [56]. In CRC metastasis, the lncRNA RPPH1 is transported via exosomes to macrophages, mediating M2 polarization and promoting metastasis [57]. Additionally, ac4C modification of KIF23 mRNA, catalyzed by NAT10, has been found to promote colorectal cancer cell progression [58].

These diverse roles highlight the significance of ncRNAs in CRC progression and their potential as therapeutic targets. Table 2 summarizes significant ncRNAs involved in CRC progression, including lncRNAs, miRNAs, and circRNAs. It highlights their mechanisms of action and potential clinical implications, showcasing their contributions to various aspects of cancer biology such as metastasis, proliferation, and chemoresistance.

#### 2.2.1. Recent scRNA-Seq Studies Revealing ncRNA Heterogeneity in CRC

The application of scRNA-seq to CRC research has unveiled unprecedented insights into tumor heterogeneity and ncRNA expression patterns. In a landmark study, in 2021, Wang et al. utilized single-cell RNA sequencing to analyze the heterogeneity in gene expression and regulatory networks across different consensus molecular subtypes (CMSs) of CRC [63]. Their study revealed distinct pathway activities and cell–cell communication patterns among CMSs, highlighting the complex interplay between tumor cells and the immune microenvironment. However, this study primarily focused on protein-coding genes and did not extensively explore non-coding RNA heterogeneity.

Advancing the field further, Xie et al. in 2022 conducted a single-cell transcriptome analysis that revealed both heterogeneity and convergence in the tumor microenvironment of CRC [64]. They identified 8 major cell types and 25 subgroups derived from tumor, para-tumor, and peripheral blood samples, providing a more comprehensive view of the CRC ecosystem.

Also in 2023, Ke et al. explored the dynamic heterogeneity of CRC during progression using eight scRNA-seq datasets [65]. They utilized the Milo algorithm to reveal differential abundance of cell clusters during progression and employed the Palantir algorithm to align cells along differentiation trajectories [66,67]. This study highlighted the enrichment of immunosuppressive Treg, myeloid cells, and fibrotic cells during CRC progression.

Addressing this gap, Mihaljevic et al. in 2024 conducted a comprehensive analysis of long non-coding RNAs (lncRNAs) in CRC using single-cell transcriptomics [10]. They analyzed 49,436 single cells from 29 CRC patients, demonstrating that lncRNAs exhibit significantly higher cell type specificity compared to protein-coding genes. Their groundbreaking work identified 996 lncRNAs strongly enriched in epithelial cells, with 98 of these differentially expressed in tumor samples compared to normal controls. This study not only provided a panoramic view of epithelial lncRNAs in CRC but also validated the functional involvement of two specific lncRNAs, CASC19 and LINC00460, in CRC disease progression using CRISPRi technology.

Further advancing the field, Li et al. in 2024 integrated short- and long-read scRNA-seq of CRC samples to construct an isoform-resolution CRC transcriptomic atlas [68]. This innovative approach allowed for the identification of 394 dysregulated transcript structures in tumor epithelial cells, including 299 resulting from various combinations of splicing events. Their work characterized genes and isoforms associated with epithelial lineages and subpopulations exhibiting distinct prognoses, uncovering 31,935 isoforms with novel junctions.

Cell type-specific ncRNA expression patterns in CRC tumors have emerged as a critical aspect of tumor biology. Mihaljevic et al.’s work in 2024 was particularly illuminating in this regard, showing that lncRNAs are more cell type-specific than protein-coding genes in CRC [10]. This finding suggests that targeting cell type-enriched lncRNAs could offer novel therapeutic strategies that exploit the unique vulnerabilities of specific CRC cell populations, particularly epithelial cells.

The importance of ncRNAs in CRC is further underscored by studies focusing on their roles in drug resistance and as potential biomarkers. For instance, a 2023 review by Luo et al. highlighted the emerging roles of various ncRNAs, including microRNAs, lncRNAs, and circular RNAs, in mediating resistance to oxaliplatin in CRC [54]. This review emphasized the potential of ncRNAs as liquid biopsy biomarkers for CRC, opening new avenues for personalized medicine approaches.

These studies have revealed unprecedented insights into the cell type-specific expression patterns of ncRNAs, their roles in tumor heterogeneity, and their potential as therapeutic targets and biomarkers. As the field continues to evolve, integrating these findings with spatial transcriptomics and proteomics data will likely yield even more comprehensive insights into the complex biology of CRC and pave the way for novel diagnostic and therapeutic strategies.

#### 2.2.2. Potential of Single-Cell Derived ncRNA Signatures for CRC Diagnosis and Prognosis

The potential of ncRNAs as biomarkers for colorectal cancer CRC diagnosis and prognosis has gained significant attention in recent years. Yamada et al. (2018) laid the groundwork by comprehensively identifying long non-coding RNAs in CRC, highlighting their importance in colorectal carcinogenesis [69].

Building on this, recent studies have demonstrated the prognostic value of ncRNA signatures in CRC. A seven-lncRNA signature was established to predict CRC patient prognosis, showing powerful discrimination ability and serving as an independent biomarker [70]. This signature outperformed traditional clinicopathological factors in predicting patient outcomes, highlighting the potential of ncRNA-based biomarkers in CRC management.

Similarly, Wang et al. (2017) identified a four-lncRNA signature (SPRY4-IT1, Loc554202, LINC01133, and RP11-727F15.13) that effectively predicted overall survival in CRC patients [71]. This signature was validated in multiple cohorts and demonstrated superior prognostic accuracy compared to conventional markers.

These findings underscore the potential of single-cell derived ncRNA signatures for CRC diagnosis and prognosis. The stability of ncRNAs in stool and blood further enhances their potential as non-invasive biomarkers for early CRC detection [72]. As scRNA-seq technology advances, it promises to uncover more refined, cell type-specific ncRNA signatures, potentially improving the accuracy of CRC diagnosis and prognosis prediction.

#### 2.2.3. Therapeutic Targeting of Non-Coding RNAs in CRC

Single-cell analysis has provided crucial insights into the potential of ncRNAs as therapeutic targets in CRC. Recent studies have revealed the extensive involvement of ncRNAs in CRC progression, including drug resistance and metastasis [73]. Mihaljevic et al. (2024) identified cell type-specific lncRNAs in CRC and validated the functional involvement of two specific lncRNAs, CASC19 and LINC00460, in CRC disease progression using CRISPRi technology [10]. These findings suggest that targeting cell type-enriched lncRNAs could offer novel therapeutic strategies that exploit the unique vulnerabilities of specific CRC cell populations.

The therapeutic potential of ncRNAs in CRC is further underscored by their roles in chemoresistance. Luo et al. (2023) highlighted the involvement of various ncRNAs, including microRNAs, lncRNAs, and circular RNAs, in mediating resistance to oxaliplatin in CRC [54]. Targeting these ncRNAs could potentially overcome drug resistance and improve treatment outcomes.

Moreover, the stability of ncRNAs in bodily fluids enhances their potential as both therapeutic targets and biomarkers. Lulli et al. (2022) noted that circulating ncRNAs have become a new source of non-invasive cancer biomarkers for CRC diagnosis, prognosis, and predicting drug therapy response [4]. This dual potential as biomarkers and therapeutic targets makes ncRNAs particularly attractive for personalized medicine approaches in CRC management.

However, challenges remain in the clinical application of ncRNA-based therapeutics, including issues of specificity, delivery, and tolerability [74]. Ongoing research is focused on developing innovative approaches to overcome these hurdles, such as improved delivery systems and more precise targeting strategies based on single-cell insights.

As the field advances, integrating single-cell transcriptomics with spatial information and functional studies will likely yield even more refined therapeutic strategies targeting ncRNAs in CRC, potentially leading to more effective and personalized treatment options for patients.

## 3. Analyzing Single-Cell Sequencing for CRC

Single-cell transcriptomics has been considered a revolutionary approach in the field of omics. Whole tissue (WT) transcriptomics has already been developed and has helped scientists understand transcription, translation, and other cellular functions. WT transcriptomics has been widely used to identify genes associated with disease, diagnosis or prognosis. Many of these genes have been identified as biomarkers of chronic, infectious, and autoimmune diseases, and cancers. While WT transcriptomics has been known to identify disease-specific patterns, single-cell transcriptomics has the power to identify cell type-specific patterns. Single-cell transcriptomics includes ligation of a unique molecular identifier (UMI) to the transcriptome of each cell. Several studies have identified CRC-associated ncRNAs, but more knowledge gaps exist in identifying cell type-specific ncRNA expression patterns [75,76,77,78]. Several previously published studies have identified several different cell populations in scRNA analyses. The molecular signatures of each cell population are usually different from others; thus, identifying cell type-specific signatures could be helpful in identifying biomarkers for diagnosis, prognosis, and novel therapeutic targets. Such cell type-specific patterns are usually lost when WT-RNA seq is performed because WT-RNA seq usually exhibits averaged expression of RNA extracted from all cells in a sampled tissue. Thus, scRNA-seq is not only a recent scientific development, but also a clinical need of the hour.

Recent developments in single-cell, next-generation sequencing (NGS), and long-read sequencing (LRS) technologies have revolutionized the fields of genetics, molecular biology, pathology, and medicine. However, this has also emerged as a challenge for life scientists from all specialties. The data produced by these technologies are big data and require specialized tools to provide insights [79,80]. Usually, life scientists are unaware of these tools, and thus, new specialties of big science, big biology, and bioinformatics have been founded. Several bioinformaticians, big biologists, and big scientists have developed pipelines for the analysis of scRNAs, NGS, and LRS. Many of these pipelines have been cited thousands of times and applied in basic and translational science. Some of these tools and pipelines include abyss, bamtools, bedtools, burrows wheel aligner or BWA, galaxy, genome analysis toolkit or GATK, miniknow, samtools, and STAR, etc., [81,82,83,84,85,86]. These tools and pipelines have become popular among scientists for NGS and LRS analysis. With the advent of scRNA-seq, new tools and pipelines have been developed for scRNA analyses. The scRNA-tools website currently tracks approximately 1800 tools. Some of these tools and pipelines include the cell ranger, scanpy, and Seurat [87,88,89,90,91,92,93]. Although each of these tools has been widely used and has unique features, but Seurat remains most popular among them.

We briefly described the scRNA analysis steps with a conceptual explanation and appropriate commands using cell ranger and Seurat below:

### 3.1. Preprocessing with Cell Ranger

The first step in scRNA analysis is preprocessing of the binary base calls (BCL). This preprocessing step includes the conversion of BCLs into the FASTQ format. BCL outputs are generated by high-end sequencing systems and must be converted into FASTQ formats when using open-source pipelines for analysis. Several bioinformatic tools exist to convert BCL to FASTQ, including BCL Convert, DigestiFlow, and DRAGON, but we recommend cell ranger when working with scRNA experiments [88,94]. The mkfastq command of the cell ranger can be used for this purpose. This command converts a BCL file into a FASTQ file.

Once FASTQ files are prepared, the next step is the alignment of sequencing reads to a reference genome/transcriptome. Once reads are mapped, any PCR duplicates and/or low base quality reads should be removed. The next step is the generation of a gene expression matrix. All of these steps can be performed using a single count command in the cell ranger. Alternatively, reads can be aligned to a reference transcriptome, and duplicates and low base quality reads can be removed using GATK [85,95,96]. The output of these cell ranger steps includes raw and filtered feature matrices, quality control (QC) reports, BAM file, and one cloupe file. The Loupe browser visualizes the cloupe file and can be used to understand the preliminary results. Bam file can be used to visualize alignments in an alignment viewer, such as IGV [97]. Raw and filtered feature matrices can be used in subsequent analyses. Although a raw matrix can be used, a filtered feature matrix is recommended to obtain a high-quality gene-cell expression matrix.

### 3.2. Data Processing in SEURAT

The cell-ranger output matrix can be imported into Seurat using the Read10X function [87]. The imported matrix should then be converted into an R-compatible format known as SeuratObject. Any outliers in the UMI count, gene count, and mitochondrial percentage should then be removed from SeuratObject. A new SeuratObject is then created without any outliers (FilterData()) and log normalized (NormalizeData()) to stabilize variance by scaling UMI counts across cells [98]. A normalized object should then be examined for the most informative genes using the FindVariableFeatures() function. As UMI counts across cells are already normalized, gene expression values are standardized using the ScaleData() function to remove batch effects and any technical biases.

### 3.3. Dimensionality Reduction and Cluster

After a SeuratObject is processed by all of the above steps, the next step is to look into the data insights and patterns. While scRNA experiments may involve thousands of cells and thousands of genes, it is challenging to identify cells and genes of interest. Mathematical methods of dimensionality reduction have the power to identify patterns in data and perform significant steps in scRNA analysis. The first dimensionality reduction step includes principal component analysis (PCA). PCA identifies highly variable cells and genes in the SeuratObject (RunPCA()). These highly variable cells and genes are then clustered using a k-nearest neighbor (KNN) graph method. Two Seurat commands, FindNeighbors() and FindClusters(), are used sequentially at this stage. After neighbors and clusters are identified in the selected highly variable group, UMAP or t-SNE methods are used to visualize clustering (RunUMAP() or RunTSNE()).

#### Differential Expression

In the first step, after the clusters are visualized, each cluster was identified with a specific cell type. This is performed using the FindAllMarkers() function for each cluster. This function generates differentially expressed genes in each cluster. These differentially expressed genes are then used to identify cell types. Once the cell type is identified, this function can be used to understand the differential expression among CRC and control cells.

### 3.4. Downstream Analysis

Once differentially expressed genes among each cell cluster are identified, it is important to interpret the meanings of these differentially expressed genes. This can be performed using pathway analysis, which can be performed using enrichment analysis tools such as Enrichr or Enrich-KG [99,100,101]. Enrichment analysis has the power to identify molecular signatures in ncRNA and CRC cells, and thus can be used to identify early activated pathways in ncRNAs, efficacy of newly developed drugs on ncRNAs, and prognosis in patients for ongoing therapies.

## 4. Single-Cell Bioinformatics Resources for ncRNA Analysis in CRC

### 4.1. Databases and Resources for CRC-Specific ncRNA Data

Several databases have been developed to store and analyze ncRNA data specific to colorectal cancer. The Cancer Genome Atlas (TCGA) provides comprehensive genomic data, including ncRNA expression profiles, for CRC patients [75]. LncRNADisease, CRlncRNA, and Lnc2Cancer databases offer information on lncRNAs associated with CRC [76,77,78,79,80]. For miRNAs, miRCancer and OncomiRDB provide curated data on miRNA involvement in CRC [81,82]. These resources are essential for researchers studying ncRNAs in CRC at the single-cell level.

### 4.2. Computational Methods for ncRNA Identification and Characterization in Single-Cell Data

Single-cell RNA sequencing (scRNA-seq) has revolutionized our ability to study ncRNAs in CRC at cellular resolution [9]. Sequence-based approaches, such as those implemented in tools like BLAST (v2.14.0) and INFERNAL (v1.1.4), are used to identify known ncRNAs [83]. Structure-based methods, including RNAfold and IPknot, predict secondary structures of ncRNAs, which can provide insights into their functions [84]. Machine learning and deep learning applications, such as LncDC and lncRNAnet, are increasingly used to predict ncRNA functions and interactions [12,85,86]. These methods can be modified to be applied to single-cell data as well.

### 4.3. Integration of Multi-Omics Single-Cell Data and Differential Expression Analysis

Integration of multi-omics data at the single-cell level is crucial for understanding ncRNA functions in CRC. Tools like MOFA+, scMVAE, and scAI enable the integration of scRNA-seq data with other omics data types [87]. Differential expression analysis of ncRNAs at single-cell resolution can be performed using methods such as MAST or scDEA, which account for the unique characteristics of single-cell data, including dropout events and technical noise [15,16].

### 4.4. Functional Enrichment, Pathway Analysis, and Network-Based Methods

Functional enrichment and pathway analysis tools like clusterProfiler and Ingenuity Pathway Analysis (IPA) can be applied to single-cell ncRNA data to infer biological functions and pathways associated with specific ncRNAs in CRC [88,89]. Network-based methods, such as WGCNA and GeneMANIA, can be used to construct regulatory networks involving ncRNAs and mRNAs in CRC, revealing potential functional interactions [90,91,92,93].

### 4.5. Survival Analysis and Prognostic Models

Single-cell ncRNA expression data can be used to develop prognostic models for CRC [94]. Methods like Cox proportional hazards regression and random survival forests can be applied to identify ncRNA signatures associated with patient survival [95]. These approaches have the potential to reveal cell type-specific ncRNA biomarkers with prognostic value in CRC.

## 5. Challenges Single-Cell Analysis of ncRNAs in CRC

**Figure 2:**
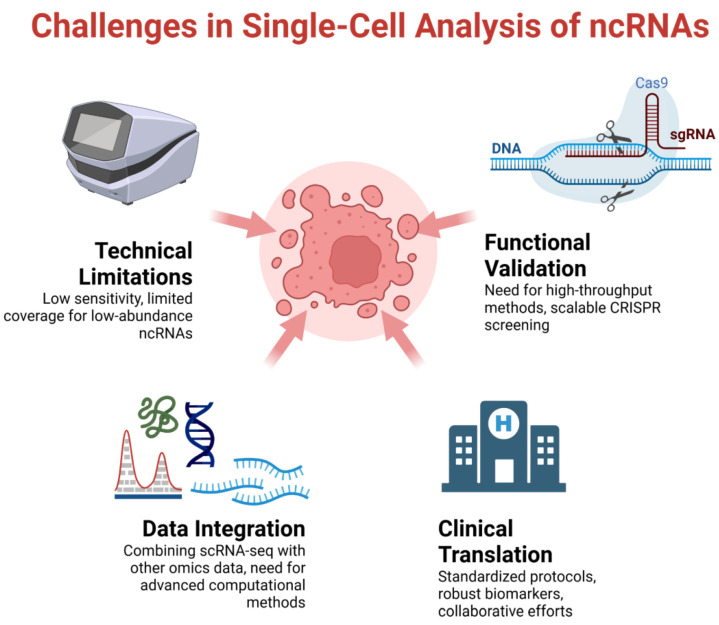
Key challenges in single-cell analysis of non-coding RNAs in colorectal cancer.

While single-cell analysis of ncRNAs in CRC has yielded promising results, several challenges remain in translating these findings to clinical applications (Figure 2).

### 5.1. Technical Limitations

Improving the sensitivity and coverage of scRNA-seq for detecting low-abundance ncRNAs remains a challenge [9,96,97]. Future technological advancements, such as improved library preparation methods and sequencing technologies, will be crucial in addressing this issue.

### 5.2. Functional Validation

High-throughput methods for validating the functions of ncRNAs identified through scRNA-seq are needed [98,99]. The development of scalable CRISPR-based approaches for functional screening of ncRNAs in single cells will be essential for moving from descriptive to mechanistic studies.

### 5.3. Integration with Other Data Types

Combining scRNA-seq data with other omics data (e.g., DNA methylation, chromatin accessibility) at the single-cell level will provide a more comprehensive understanding of ncRNA regulation in CRC [100]. The development of multi-omics single-cell technologies and advanced computational methods for data integration will be crucial in this regard.

### 5.4. Clinical Translation

Developing standardized protocols for single-cell analysis of clinical samples and establishing robust ncRNA-based biomarkers for routine clinical use are ongoing challenges [101]. Collaborative efforts between researchers, clinicians, and industry partners will be essential to overcome these hurdles and bring single-cell ncRNA-based diagnostics and therapeutics to the clinic.

## 6. Discussion

### 6.1. Revolutionizing ncRNA Research in CRC Through Single-Cell Sequencing

Single-cell RNA sequencing has transformed our understanding of ncRNA biology in colorectal cancer (CRC), offering unprecedented insights into tumor heterogeneity, cell type-specific expression patterns, and potential therapeutic targets. This review highlights the crucial role of bioinformatics approaches in analyzing and interpreting the vast amount of data generated by single-cell studies of ncRNAs in CRC.

### 6.2. Integration of Computational Methods

The integration of computational methods, including sequence-based approaches, structure-based methods, and machine learning applications, has enabled the identification and characterization of ncRNAs at unprecedented resolution. Sequence-based methods, such as k-gram analysis and multivariate mutual information, offer high-throughput analysis capabilities and the ability to identify novel ncRNAs, although they can be limited by sequence quality and annotation accuracy. Structure-based methods, including minimum free energy (MFE) approaches and probabilistic models, provide valuable insights into ncRNA function and interactions but can be computationally intensive [83]. Machine learning techniques, such as support vector machines (SVMs) and deep neural networks, have shown promise in identifying and classifying ncRNAs, though they often require large training datasets and may be prone to overfitting [83,102].

### 6.3. DNA Analysis and Epigenetic Considerations

Complementing RNA-based methods, computational approaches for DNA analysis, particularly those focusing on nucleosome positioning and DNA methylation, have become increasingly important in understanding the epigenetic landscape of CRC [103,104]. Tools such as NuPoP, nuScore, and N-score utilize various features to predict nucleosome positions, offering a more comprehensive view of the epigenetic factors influencing ncRNA expression and function in CRC [105,106].

### 6.4. Future Perspectives and Challenges

As the field advances, the combination of single-cell transcriptomics with spatial information, multi-omics data integration, and artificial intelligence will likely yield even more refined insights into ncRNA function in CRC. The development of sophisticated computational tools for differential expression analysis, functional enrichment, and regulatory network construction will be crucial for unraveling the intricate roles of ncRNAs in CRC biology.

The potential of ncRNAs as diagnostic and prognostic biomarkers, as well as therapeutic targets, has been underscored by recent single-cell studies. The identification of cell type-specific ncRNA signatures and their association with clinical outcomes opens new avenues for personalized medicine approaches in CRC management.

However, significant challenges remain in translating these findings into clinical applications. Overcoming technical limitations in single-cell sequencing, improving computational methods for data analysis, and validating ncRNA targets through functional studies will be critical steps in advancing the field.

## 7. Conclusions

The integration of single-cell transcriptomics with advanced bioinformatics approaches has ushered in a new era in ncRNA research in CRC. The rapid progress in single-cell bioinformatics approaches for studying ncRNAs in CRC underscores the importance of continued research in this area. As our understanding deepens, the potential for ncRNA-based precision oncology in CRC management becomes increasingly promising, offering hope for improved patient outcomes in the future.

By continuing to refine these methods and translate findings into clinical applications, we can work towards more effective strategies for CRC diagnosis, prognosis, and treatment, ultimately improving the lives of patients affected by this devastating disease. The ongoing development of sophisticated computational tools and the integration of multi-omics data will be crucial in unraveling the complex roles of ncRNAs in CRC biology, paving the way for significant advancements in personalized medicine and targeted therapies.

## Figures and Tables

**Table 1:** Types and Functions of Non-Coding RNAs (ncRNAs) in Cellular Processes. This table summarizes the major types of non-coding RNAs (ncRNAs), including long non-coding RNAs (lncRNAs), microRNAs (miRNAs), and circular RNAs (circRNAs), along with specific examples and their respective roles in cellular processes. The table highlights the diverse functions of these ncRNAs in gene regulation, cell differentiation, proliferation, migration, and their involvement in various physiological and pathological conditions.

Type	ncRNAs	Role in the Cell	Reference
Long Non-Coding RNAs (lncRNAs)	HOTAIR	Key regulator of body plan during development and cellular Identity.	[26,27]
	MALAT1	Involved in the endothelial cell function.	[28]
	BANCR	Works as a regulatory molecule for cell, proliferation, migration and expression.	[29]
	TUG1	Acts as a tumor suppressor/regulation on cell apoptosis.	[30]
	NALT 1	Particularly involved in the immune response and inflammation.	[31]
	FEZF1-AS1	Plays an important role in gene regulation, cell cycle regulation, and epigenetic modifications leading to the major participant in cancer cell proliferation and migration.	[32]
microRNAs (miRNAs)	miR-21	Involved in the post-transcriptional regulation.	[33]
	miR-31	Involved in cell’s normal physiological processes and plays an important role in the embryonic implantation, development of bone tissues, and immune system functionality.	[34]
	miR-34a	Involved in the differentiation of NSCs.	[35]
	miR-181a-5p	Involved in regulation of cell differentiation, osteoblasts, immune, hematopoietic cells, chondrocytes and adipocytes. Also plays a key role in cardiac pulmonary and vascular abnormalities.	[36]
	miR-135b	Plays a major role in cell proliferation, differentiation and viral infections. Also can act as an oncogenic nd tumor suppressor.	[37]
	miR-126	Involvement in regulation of VEGF. Knockdown of this miRNA can lead to hemorrhage and loss of vascular integrity during embryonic development.	[38]
	KIF23	Kinesin Family Member 23 plays an important role in cell division and intracellular transport.	[39]
Circular RNAs	circ_001569	Gene regulators, act as natural miRNA sponges.	[40]
	circHIPK3	Regulation of cell growth by spooning with the miRNAs.	[41]
	circCCDC66	Regulates various cell functions also involve in the CTRC, Osteoarthritis progression, and many other diseases.	[42]

NSCs: Neural stem cells, VEGF: Vascular Endothelial Growth Factor.

**Table 2:** Key Non-Coding RNAs and Their Roles in Colorectal Cancer. The table emphasizes the potential of these ncRNAs as diagnostic and prognostic biomarkers, as well as therapeutic targets in CRC management, underscoring the complexity of ncRNA involvement in CRC and promising avenues for future research and clinical applications.

Study	ncRNA	Mechanism	Implications	Statistical Limitations	Limitations	Reference
CRC Metastasis	HOTAIR, MALAT1	MALAT1 promotes tumor growth and metastasis by regulating gene expression related to cancer progression, including enhancing β-catenin signaling and affecting downstream target genes like RUNX2.	High expression levels of HOTAIR and MALAT1 are associated with liver metastasis and poor survival outcomes in CRC patients, suggesting their potential as prognostic biomarkers.	Expression is inconsistent, i.e., different tumor types might show the same discrete response. Moreover, there is no significant relationship between survival and the lncRNA due to the lack of long-term patient follow-ups.	RNA can degrade over time. The studied populations are small. Designs are single-centered leading to an increased risk of technical variability.	[18,19]
	RPPH1	Transported via exosomes to macrophages	Mediates M2 polarization of macrophages, promoting CRC metastasis	The pathological and physiological role of RPPH1 is very little known. No noticeable change was found at different stages of the tumor leading to the lack of a significant link between clinical features and the miRNA.	Rt PCRs using various probes are mostly used to detect the miRNAs. Differentiation between the different PCR products using SYBR-Green I is challenging. Increased overall cost.	[57]
	lncRNA FEZF1-AS1	Promotes epithelial–mesenchymal transition	Activates invasion and metastasis in CRC	The association of FEZF1-AS1 with PKM2 is less known. Further research is required to develop inhibitors specifically targeting FEZF1-AS1/PKM2 signaling. FEZF1-AS1 inhibits the apoptosis of cancer cells in CRC which is consistent with other cancer types.	Low expression of FEZF1 in CRC. Precise cites are not known. The association of FEZF1 with CIN requires further investigation.	[52]
CRC Proliferation	miR-21, miR-31	Positive regulators of colon carcinoma cells, promoting proliferation and invasion	Potential biomarkers for CRC diagnosis and prognosis	The expression of miR-21 and miR-31 is dependent on the tissue type.	Rt PCR using various probes such as SYBR-Green I can lead to an inaccurate or false positive quantification due to its less specificity.	[22]
	TUG1	TUG1 is a direct transcriptional target of p53, influencing cell proliferation through the PRC2 complex	TUG1 may serve as a biomarker and therapeutic target due to its association with tumor progression and metastasis	TUG1 can be associated with various tumor types and work as oncogenes or oncogene suppressors.	Although TUG1 is a very promising prognostic marker its clinical utility is uncertain.	[47]
	circ_001569	Sponges miR-145 to regulate E2F5, BAG4, and FMNL2	Promotes CRC proliferation and invasion; upregulated in CRC tissues	A thorough analysis of circRNAs in CRC has not been reported so far. The circRNAs biogenesis is elusive.	High-throughput sequencing methods and bioinformatic tools need to be developed to detect circRNAs.	[56]
	CRNDE, miR-181a-5p	Overexpression of CRNDE suppresses miR-181a-5p, which targets β-catenin and TCF4 and increase proliferation.	The regulation of Wnt/β-catenin by miR-181a-5p’s is disturbed in CRC due to over and less expression of CRNDE. Upregulation of CRNDE promotes proliferation.	The molecular mechanism of CRNDE in the progression of CRC remains clear. Many other molecular factors can cause CRC progression and chemoresistance. No direct evidence to confirm if the signaling activity of Wnt/β-catenin is required to regulate cell proliferation and chemoresistance of CRC by miR-181a-5p and CRNDE.	Lack of detailed online database. RNAs are sensitive to handle, and a minor mishandling can lead to their degradation.	[59]
	miR-135b	Targets APC and TGFBR2, leading to increased proliferation and decreased apoptosis	Potential oncogene and therapeutic target in CRC	Interactions of miRNAs (miR-135b, miR-31, miR-96 and miR-21) with their potential targets require further studies.	Small datasets. Less statistical significance. The complexity of CRC in living organisms cannot always be replicated by online databases.	[45]
CRC Progression	NALT1	Sponges miR-574-5p to upregulate PEG10	Promotes CRC proliferation, migration, and invasion; high expression correlated with advanced cancer stage	The role of NALT1 in CRC progression remains unclear. The regulation network for NALT1, in early-stage CRC, and the association of PEG10 with the development of NALT1-mediated CRC needs further investigation.	Lack of detailed online database. RNAs are sensetive to handle.	[55]
	miR-149	Methylation of miR-149 contributes to the growth of CRC by targeting the transcription factor Sp1.	Ssignificantly decreased miR-149-5p in GPC+ exosomes from tumor tissues and plasma of CRC ad compared to that of healthy controls.	The function of miR-149-5p in drug sensitivity lacks comprehensive statistical validation.	Discrepancies can be caused by data integrations from multiple sources.	[60]
	KIF23 mRNA	Interacts with ac4C modification	Promotes colorectal cancer cell progression; ac4C catalyzed by NAT10	Overexpression is common in various tumor types. Large-scale studies are required for a thorough understanding of heterogeneity, and RNA modifications and to develop targeted therapies for CRC.	Various challenges exist for the application of RNA modification methods into clinical use.	[58]
CRC Growth	circHIPK3, circCCDC66	circHIPK3 sponges miR-7; circCCDC66 acts as a sponge for miR-3140	Potential therapeutic targets and biomarkers for CRC	Multiple binding sites of circRNAs for a variety of miRNAs explain the complexity of their role in CRC malignancy. The regulatory gene network of circRNA-miRNA in progression and treatment investigations needs to be further investigated.	Various binding sites of circRNA can be challenging.	[24,25]
MYC Expression Promotion	CCAT2	Regulation of MYC and WNT signaling pathways	Potential biomarker and therapeutic target in CRC	A complicated network leading toward CCAT2-provoked metastatic phenotype needs to be identified.	The rs6983267 allele has a very minimal effect on the expression levels of CCAT2 so better experimental designs are needed for expression detection.	[44]
Chemoresistance	miR-34a	Targets SIRT1 and TGF-β/Smad4 pathway	Low expression associated with oxaliplatin resistance; overexpression enhances chemosensitivity	Treatment failure is common in various patients due to acquired or congenital resistance. Molecular mechanisms behind oxaliplatin resistance needs to be further investigated.	Limitated efficiently for obtaining highly abundant and quality exosomes. The loading capacity of ncRNAs delivered by endogenous exosomes is very less.	[54]
	CRNDE, miR-181a-5p	When CRNDE is knocked down the miR-181a-5p is overexpressed leading to chemoresistance.	The regulation of Wnt/β-catenin by miR-181a-5p’s is disturbed in CRC due to over and less expression of CRNDE. Downregulation of CRNDE promotes proliferation.	The molecular mechanism of CRNDE in the progression of CRC remains clear. Many other molecular factors can cause CRC progression and chemoresistance. No direct evidence to confirm if the signaling activity of Wnt/β-catenin is required to regulate cell proliferation and chemoresistance of CRC by miR-181a-5p and CRNDE.	Not detailed online database. Sensetivity of RNAs to handle.	[59]
Angiogenesis	miR-126	Targets VEGF signaling	Modulates angiogenesis in CRC	Undifferentiated tumors. Lack of data on possibly related mutations such as KRAS, NRAS, and BRAF. Ineffective histological reports as compared to recent standards.	Lack of digital pathology. Discrepancies can be caused by data integrations from multiple sources (meta-analysis)	[50]
CRC Migration	BANCR	BANCR facilitate EMT (epithelial–mesenchymal transition) and promotes CRC migration.	High expression levels of BANCR are associated with advanced tumor stage.	The role of BANCR in the development, expression patterns, and functionality of CRC is less known. Lack of significant correlation between BANCR expression and clinicopathological features(gender, age, tumor location, size, invasion depth, and histological grade). Limited study on gene expression changes.	Lack of analysis of long-term survival rates. Sufficient data are not available on the precise mechanism of BANCR to influence EMT in CRC.	[61]
CRC Oncogenesis	miR-17	Promotes CRC by activating the Wnt/β-catenin and targeting P130.	High expression of miR-17 may contribute to liver metastasis in CRC	Late-stage diagnosis of CRC in the majority of patients. No association with clinicopathological features such as age, gender, and lymphatic metastasis. Analysis of the expression of miR-17 in the primary stage of CRC and liver metastases, in larger sample sizes is required to confirm conclusions.	Lack of in-depth knowledge of downstream pathways.	[62]

CRC: Colorectal Cancer, M2 polarization: Referring the macrophages polarization into M2 phenotype, PCR: Polymerase Chain Reaction, PKM2: Pyruvate Kinase M2, TUG1: Taurine Upregulated Gene 1, p53: A tumor suppressor protein, PRC2: Polycomb Repressive Complex 2 (Protein complex), E2F5: Transcription factor, BAG4: Bcl-2-associated Athanogene 4 (protein), FMNL2: Formin-Like 2 (protein), Wnt/β-catenin: Signaling pathway essential in cell proliferation, progression and differentiation, CRNDE: Colorectal Neoplasia Differentially Expressed (lncRNA associated with CRC), TCF4: Transcription Factor 4, APC: Adenomatous Polyposis Coli (tumor suppressor gene), TGFBR2: Transforming Growth Factor Beta Receptor 2, GPC: Glypican, ac4C: N4-acetylcytidine (modification of RNA), NAT10: N-acetyltransferase 10 enzyme, MYC: Myelocytomatosis oncogene, WNT: Secreted glycoproteins (Wnt signaling pathway), SIRT1: Sirtuin 1, TGF-β/Smad4: Transforming Growth Factor Beta signaling pathway, Smad 4 is a transcriptional factor, VEGF: Vascular Endothelial Growth Factor, EMT: Epithelial–Mesenchymal Transition.

## Data Availability

The data presented in this study are available in this article.
